# PANoptosis: bridging apoptosis, pyroptosis, and necroptosis in cancer progression and treatment

**DOI:** 10.1038/s41417-024-00765-9

**Published:** 2024-03-29

**Authors:** Jie Gao, Anying Xiong, Jiliu Liu, Xiaolan Li, Junyi Wang, Lei Zhang, Yao Liu, Ying Xiong, Guoping Li, Xiang He

**Affiliations:** 1 Laboratory of Allergy and Precision Medicine, Chengdu Institute of Respiratory Health, the Third People’s Hospital of Chengdu, Affiliated Hospital of Southwest Jiaotong University, Chengdu, 610031 China; 2 Department of Pulmonary and Critical Care Medicine, Chengdu third people’s hospital branch of National Clinical Research Center for Respiratory Disease, Affiliated Hospital of ChongQing Medical University, Chengdu, 610031 China; 3 National Center for Respiratory Medicine, National Clinical Research Center for Respiratory Disease, State Key Laboratory of Respiratory Disease, Institute of Respiratory Health, The First Affiliated Hospital of Medical University, Guangzhou, Guangdong, 510120 China; 4 Department of Pulmonary and Critical Care Medicine, Sichuan friendship hospital, Chengdu, 610000 China

**Keywords:** Cancer microenvironment, Tumour immunology, Cell biology, Targeted therapies

## Abstract

This comprehensive review explores the intricate mechanisms of PANoptosis and its implications in cancer. PANoptosis, a convergence of apoptosis, pyroptosis, and necroptosis, plays a crucial role in cell death and immune response regulation. The study delves into the molecular pathways of each cell death mechanism and their crosstalk within PANoptosis, emphasizing the shared components like caspases and the PANoptosome complex. It highlights the significant role of PANoptosis in various cancers, including respiratory, digestive, genitourinary, gliomas, and breast cancers, showing its impact on tumorigenesis and patient survival rates. We further discuss the interwoven relationship between PANoptosis and the tumor microenvironment (TME), illustrating how PANoptosis influences immune cell behavior and tumor progression. It underscores the dynamic interplay between tumors and their microenvironments, focusing on the roles of different immune cells and their interactions with cancer cells. Moreover, the review presents new breakthroughs in cancer therapy, emphasizing the potential of targeting PANoptosis to enhance anti-tumor immunity. It outlines various strategies to manipulate PANoptosis pathways for therapeutic purposes, such as targeting key signaling molecules like caspases, NLRP3, RIPK1, and RIPK3. The potential of novel treatments like immunogenic PANoptosis-initiated therapies and nanoparticle-based strategies is also explored.

## Introduction

Numerous studies have consistently shown that controlling cell proliferation and removing unnecessary or potentially harmful cells is essential in maintaining homeostasis for the growth and development of multicellular organisms in vivo [1]. Programmed cell death (PCD) is an effective mechanism for regulating these processes, designed to actively sculpt, control, and aid in the development and survival of the body [2]. PCD encompasses both classical apoptosis within the context of developmental and tissue homeostasis, as well as alternative forms that arise in response to exogenous or endogenous microenvironment perturbations, such as apoptosis, pyroptosis, necroptosis, ferroptosis, autophagy, and others [3].

The study of the most classical and well-defined PCD pathways of pyroptosis, apoptosis, and necroptosis, and most importantly, the interaction between these three pathways, has led to the establishment and development of the concept of ‘PANoptosis’. Evidence for PANoptosis was first provided in a study of influenza A virus (IAV)-infected macrophages, which demonstrated that activation of caspase-1, caspase-3, and caspase-8, as well as phosphorylation of MLKL, were key molecular events in pyroptosis, apoptosis, and necroptosis [4]. It is worth noting that PANoptosis has key features of the above pathways, but cannot be explained by any one of them alone. Rather, they are interconnected and maintain dynamical equilibrium by sharing a deadly protein complex, namely ‘PANoptosome’, which is thought to be a platform that can engage multiple modes of cell death [5]. As such, significant crosstalk occurs in the execution and regulation of the numerous pathways in PANoptosis.

Recent studies have focused on the complex molecular mechanisms underlying PANoptosis and its implications in human diseases, particularly cancer [6, 7]. The progression of cancer has been linked to mechanisms that allow cancer cells to bypass PANoptosis. Therefore, understanding the connection between PANoptosis and cancer may provide deeper insights into the occurrence and treatment of cancer, and may be critical in exploring new therapeutic strategies by interfering with the escape mechanism of PANoptosis in cancer cells. Additionally, within the TME, PANoptosis can combat tumor progression through local regulation and immune responses, including the killing effect of immune cells. However, studies on these mechanisms are still in their infancy and require further exploration.

In this study, we will focus on the molecular mechanisms of each PCD pathway, the significant crosstalk events present in PANoptosis, and investigate their relationship with the TME and cancer, as well as targeted therapeutic strategies.

## Mechanisms and crosstalk events in PANoptosis

### PANoptosis: apoptosis, pyroptosis, and necroptosis

**Fig. 1:**
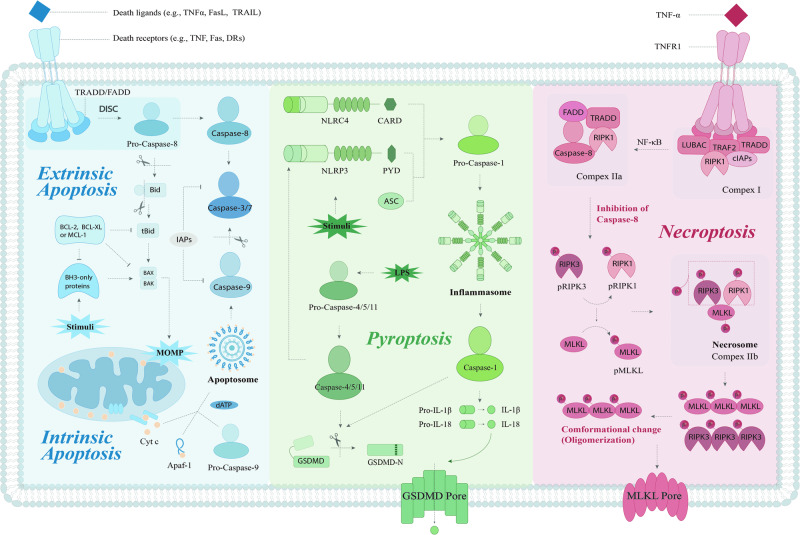
Molecular machinery of apoptosis, pyroptosis, and necroptosis. Pro-apoptotic (e.g., BAX, BAK, Bid) and anti-apoptotic factors (e.g., BCL-2, BCL-XL, MCL-1) control the intrinsic apoptosis pathway. When only BH3 proteins are activated in the pro-apoptotic members, BAX and BAK induce MOMP, leading to the release of Cyc.C and the formation of an apoptosome with Apaf-1, which in turn activates caspase-9. The extrinsic pathway, mediated mainly by death receptors and their ligands, results in the formation of the DISC, which activates pro-caspase-8. A cascade of apoptotic processes is triggered by the activation of downstream caspase-3 and -7 by both caspase-8 and -9. In the classical pathway of pyroptosis, inflammatory sensors, such as NLRP3, recruit ASC upon various stimuli to promote pro-caspase-1 activation, thereby further facilitating the assembly of the inflammasome and the activation of caspase-1. Activated caspase-1 cleaves GSDMD and pro-caspase-1, which forms the GSDMD pore and promotes the release of inflammatory factors. The nonclassical pathway involves LPS in the cytoplasm, activating caspase-4/5/11 and cleaving GSDMD to initiate pyroptosis. Necroptosis typically involves the TNF signaling pathway. Complex I, composed of TRADD, TRAF2, RIPK1, the cellular inhibitors of apoptosis (cIAPs), and the linear ubiquitin chain assembly complex (LUBAC), can promote NF-кB signaling. After that, FADD and TRADD are recruited to form complex IIa with caspase-8 and RIPK1. When caspase-8 is inhibited, RIPK1, RIPK3, and MLKL are phosphorylated successively, resulting in the formation of the necrosome (complex III). The MLKL pore is formed when MLKL oligomerizes and moves to the plasma.

To date, apoptosis, pyroptosis, and necroptosis are the three PCD pathways that have been most comprehensively studied and most fully characterized in response to cellular injury, but their molecular machinery is quite different (Fig. 1).

Apoptosis can be divided into two pathways: intrinsic and extrinsic. The intrinsic pathway occurs within the mitochondria and can be triggered by various stimuli such as toxins, hypoxia, growth factors, and viral infections [8], while the latter involves the TNF gene superfamily, which is mediated by death receptors. The intrinsic pathway is regulated by pro-apoptotic BCL-2 family members (BAK, BAX, Bid, etc.), which promote mitochondrial outer membrane permeabilization (MOMP) and release of cytochrome c (Cyt. c) [9–11]. Cyt. c then forms a complex with Apaf-1 and dATP in the cytoplasm, known as the apoptosome, which activates pro-caspase-9 [12–14]. In the extrinsic pathway, the death receptors containing death domains (DD) are involved (e.g., FADD, TRADD). These death receptors recognize specific ligands and activate the recruitment and activation of caspase-8, leading to the formation of a death-inducing signaling complex (DISC). Caspase-8 then activates executioner caspases or induces the intrinsic pathway. Both pathways converge at the activation of caspase-3 and caspase-7, which carry out proteolytic cleavage and further activation of downstream targets [15, 16].

Pyroptosis is a form of cell death mediated by the inflammasome complex, which mainly consists of a sensor, an adaptor, and a zymogen pro-caspase-1. Some types of inflammasomes have been identified, of which NLRP3 is the best-studied member. Inflammasome sensors are usually grouped into the NLRs, AIM2 (absent in melanoma 2), or pyrin, possessing the capability to assemble inflammasomes and directly or indirectly activate caspase-1 [17] when subjected to various pathogen-associated molecular patterns (PAMPs) and damage-associated molecular patterns (DAMPs). The ASC, an adaptor protein, is composed of two death-fold domains: a pyrin domain (PYD) and a caspase recruitment domain (CARD), which allow ASC to bridge and couple the upstream inflammasome sensor molecule to the effector cysteine protease caspase-1 [18, 19]. Activated caspase-1 cleaves GSDMD (gasdermin D), leading to the formation of pores in the plasma membrane and the release of cytokines IL-1β and IL-18 [20–23]. Differently, murine caspase-11 (or caspase-4 and -5 in humans) mediates noncanonical inflammasome signaling by directly recognizing Gram-negative bacterial LPS (lipopolysaccharides), triggering proteolytic activation of pore-forming GSDMD, which drives pyroptosis [24–26].

Necroptosis, as it is defined, is characterized by both necrosis and apoptosis. Although it displays morphological traits similar to necrosis, it is actually part of the apoptosis pathway, inducing ordered cell death through the activation of specific death signaling pathways [27]. The TNF/TNFR signaling pathway is a well-studied inducer of necroptosis [28, 29]. RIPK1, an upstream regulator, is thought to be a key signaling node in the TNF signal transduction pathway, actively regulating the balance between gene activation and cell death induction in the form of apoptosis and necroptosis [30]. In the absence of apoptosis, i.e., when caspase-8 activity is inhibited, the assembly of RIPK1/RIPK3 necrosome promotes programmed necrosis [31]. The necrosome subsequently phosphorylates the activation loop of the most terminal obligate effector MLKL (mixed-lineage kinase domain-like protein), leading its translocation to the plasma membrane where it disrupts membrane integrity, resulting in necroptosis [32–35].

### Crosstalk and regulation in PANoptosis

**Fig. 2:**
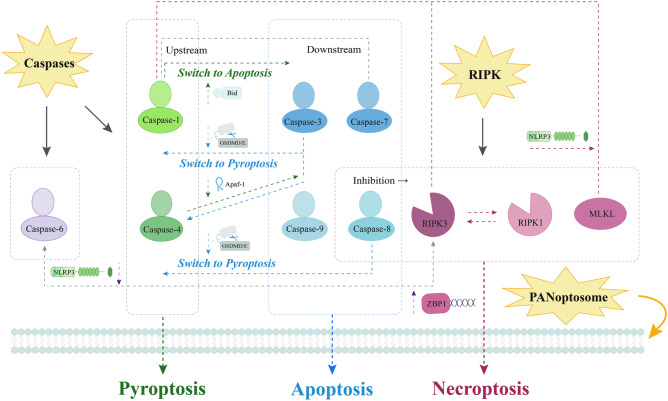
Crosstalk events in the mechanism of PANoptosis. Diverse regulatory processes in PANoptosis are mainly realized through the multiprotein complex PANoptosome. Under the regulation of the PANoptosome, a large number of crosstalk events occur, and the core of these events includes key proteins in apoptosis, pyroptosis, and necroptosis, with the Caspase family and the RIPK family playing the most prominent roles.

A growing body of research has emphasized that the apoptosis, pyroptosis, and necroptosis pathways involved in PANoptosis can be co-activated within the same cell, and it has been demonstrated that these three pathways, while operating in parallel, can cross-regulate each other mediated by a multiprotein complex known as the PANoptosome. The concept of the PANoptosome was introduced by Christgen et al. [36]. Their findings suggested direct interactions between PANoptotic molecules and the formation of the PANoptosome, and confirmed the existence of broad crosstalk events in the mechanism of PANoptosis (Fig. 2). As of present, more different species of PANoptosomes have been identified, including the ZBP1-PANoptosome, the AIM2-PANoptosome, the RIPK1-PANoptosome, and the NLRP12-PANoptosome, as reported in great detail [6, 37–39].

Caspases are a class of proteases with well-defined roles in apoptosis [40], as well as central players in pyroptosis. Several studies have clearly revealed the critical role of caspases-mediated activation and inactivation of gasdermin family of proteins, as well as their important role in cell membrane lysis and subsequent inflammatory cell death [41]. Moreover, caspases converge in inflammasome activation, cell death, and innate immunity [42], providing strong evidence for the existence of crosstalk events in PANoptosis (Table 1). While Caspase-1 has long been considered a key component in mediating pyroptosis, it also functions via the Bid-caspase-9-caspase-3 axis in cells lacking GSDMD to initiate apoptosis [43]. Caspase-3, the downstream executioner of apoptosis, can target the plasma membrane to induce secondary necrosis or pyroptosis after cleavage of the GSDMD-related protein DFNA5 [44]. Meanwhile, GSDME/DFNA5 has also been shown to switch TNF- or chemotherapy drugs-induced caspase-3-mediated apoptosis to pyroptosis [45], indicating inevitable crossing lines between apoptosis and inflammation-associated pyroptosis. Moreover, this crosstalk can be bidirectional, as verified in apoptosis, where caspase-3 can cleave the GSDMD to potentially block pyroptosis via a different site from the inflammatory caspases that inactivate the protein [46].

**Table 1:** Classification of major caspases in PANoptosis.

Caspases	Expressible species	Classification		Domain structure
		Inflammatory or apoptotic	Initiator or effector	
Caspase-1	Hu, Ms	Inflammatory	Initiator	
Caspase-4	Hu	Inflammatory	Initiator	
Caspase-5	Hu	Inflammatory	Initiator	
Caspase-11	Ms	Inflammatory	Initiator	
Caspase-2	Hu, Ms	Apoptotic	Initiator	
Caspase-8	Hu, Ms	Inflammatory/apoptotic	Initiator	
Caspase-9	Hu, Ms	Apoptotic	Initiator	
Caspase-10	Hu	Apoptotic	Initiator	
Caspase-3	Hu, Ms	Apoptotic	Effector	
Caspase-6	Hu, Ms	Apoptotic	Effector	
Caspase-7	Hu, Ms	Apoptotic	Effector	

*Hu* human, *Ms* mouse, CARD caspase activation and recruitment domain, *DED* death effector domain, *L* large domain, *S* small domain.

The caspases involved in PANoptosis can be broadly categorized into inflammatory caspases and apoptotic caspases. Inflammatory caspases mainly include caspase-1, caspase-4, caspase-5, and caspase-11, which belong to the class of initiators. Apoptotic caspases mainly include caspase-2, caspase-8, caspase-9, caspase-10 (all initiators), and caspase-3, caspase-6, caspase-7 (all executors). These caspases have specialized domain structure and can be expressed in human or mouse.

Similarly, caspase-4 is another performer in the nonclassical pathway of pyroptosis. It has been shown that caspase-4, activated in Apaf-1 pyroptosome mediated by Apaf-1 (an important player in apoptosis), can continue to cleave caspase-3, which in turn leads to GSDME-induced pyroptosis [47]. Other studies have proposed that caspase-6 is required for ZBP1-mediated NLRP3 inflammasome activation and PCD in a protease activity-independent manner. Although functionally categorized as an apoptotic executioner caspase, caspase-6 does not possess the same substrate specificity as caspase-3 and -7, but rather resembles the initiator caspase-8 and -9. Besides, during IAV infection, caspase-6 interacts with RIPK3 and facilitates the binding of RIPK3 and ZBP1, thus enhancing ZBP1-mediated inflammasome activation and inflammatory cell death [48]. Caspase-7, identified as a new substrate of caspase-1, has emerged as an interesting factor. It has been shown that the caspase-1 inflammasome plays an upstream role in caspase-7 activation in vivo, and this activation has been validated in vitro and in knock-out macrophages [49].

Notably, caspase-8 is not only a key component of the exogenous apoptosis pathway, but also serves as a common molecular switch for apoptosis, necroptosis, and pyroptosis, functioning as a signaling hub for the overall mechanism [50]. Its role in inflammasome activation and pro-IL-1β processing is being progressively discovered. For instance, it has been observed that in macrophages, BAX/BAK-induced apoptosis triggers the activation of caspase-8 and aids in the maturation of IL-1β, a central pro-inflammatory cytokine [51]. Other studies have indicated that caspase-8 is an essential component required for both transcriptional priming and activation of typical and atypical NLRP3 inflammasomes in mice [52–54], and that its activation during TAK1 inhibition can lead to GSDMD and GSDME cleavage in murine macrophages [55, 56]. Caspase-8 mediates apoptosis induced by death receptors and inhibits necroptosis mediated by RIPK3 and MLKL. Mutant caspase-8 induces the formation of ASC specks, and caspase-1-dependent cleavage of GSDMD and caspase-3 and 7 in MKKL-deficient mice [57]. Research by Zhang et al. found that p90 ribosome S6 kinase (RSK) recruited into the necrosome phosphorylated pro-caspase-8 at Thr265, eliminating caspase-8 activity and leading to the occurrence of necroptosis [58]. This was also supported by another study in which caspase-8 naturally controls the RIPK3-dependent death pathways in check in addition to promoting apoptosis [59]. Additionally, Caspase-8 also played a promotional role for NLRP3 inflammasome activation, and its scaffolding function and MLKL were necessary for regulating NLRP3 inflammasome activation downstream of TLR3 [60].

Receptor-interacting protein kinases (RIPK), specifically RIPK1 and RIPK3, serve as substrates for caspase-8 and play pivotal regulatory roles in the PANoptosis signaling pathway. RIPK1 is involved in both RIPK3-dependent and non-dependent signaling pathways that lead to cell death and/or inflammation. Studies have demonstrated that RIPK1 blocks early postnatal lethality mediated by caspase-8 and RIPK3, as well as regulates FADD-caspase-8 and RIPK3-MLKL signaling [61]. Another more novel idea that has garnered attention is the finding that when the level of RIP1 exceeds approximately 1000 molecules per cell (mpc), both caspase-8 and RIPK3 can be recruited into the necrosome, resulting in the simultaneous initiation of apoptosis and necroptosis, respectively. Conversely, when a higher amount of RIP1 (>~46,000 mpc) is present, apoptosis is inhibited, and only necroptosis occurs [62]. This suggests that the level of RIP1 can point to different outcomes for the fate of cell death, mainly achieved by regulating its expression differently.

Furthermore, as the study progressed, the researchers confirmed that NLRP3 inflammasome activity is a driver of inflammation in MLKL-dependent diseases. Under certain conditions, necroptosis signaling could trigger the RIPK3-MLKL-NLRP3-Caspase-1 axis, resulting in the maturation and release of IL-1β independently of GSDMD [63–65].

## The association of PANoptosis with cancer

As of now, substantial evidence has demonstrated that the subroutines of PCD, including apoptosis, pyroptosis, necroptosis, and ferroptosis, are pivotal in tumorigenesis. Loss of control over individual or combined PCD subroutines can contribute to the development of cancer [66]. Aberrant expression of PANoptosis-related genes (PRGs), such as NLRP3, caspase-8, and TNFAIP3, has been observed in various cancer types, with remarkable mutations detected. Notably, many PRGs serve as tumor risk factors across different types of cancer. Furthermore, PANoptosis genes and PANoptosis scores have been significantly linked to patient survival in 21 and 14 cancer types, respectively [67]. In the context of cancer, increased expression of A ADAR1-p150 has been demonstrated to restrict the binding of ZBP1 and RIPK3, consequently suppressing ZBP1-mediated PANoptosis to facilitate tumor growth [68]. These findings suggest that PANoptosis may exert a positive influence on specific cancer types, and inducing PANoptosis could aid in inhibiting cancer development and progression.

### PANoptosis in respiratory cancers

The role of PANoptosis in lung cancer is currently under investigation. FADD serves as a key adaptor in PANoptosis and is one of the prominent risk factors for lung cancer. In vitro experiments have indicated that the knockdown of FADD promotes apoptosis and pyroptosis and significantly reduces the proliferative capacity of cancerous lung cells, suggesting that the identification of prognostic traits based on the FADD-regulated genes may provide a new direction for the treatment of lung cancer [69]. Moreover, TRADD, NLRC4, RIPK1, MLKL, and PSTPIP are PRGs associated with prognosis in lung adenocarcinomas (LUADs) and are essential in PANoptosis involving apoptosis and pyroptosis. Elevated expressions of TRADD, NLRC4, RIPK1, and PSTPIP2 in patients were correlated with improved prognosis, whereas elevated expressions of FADD and MLKL were linked to poor prognosis [70]. Several PANoptosis genes were significantly associated with LUAD survival [71]. This finding strongly implies the involvement of PANoptosis in the tumor microenvironment (TME) and immune regulation in LUAD.

### PANoptosis in digestive system cancers

PANoptosis is currently being extensively studied in digestive system cancers. In gastric cancer (GC), the role of PANoptosis was explored earlier and found to be closely related. Patterns of PANoptosis in GC patients were observed to predict survival and immunotherapy response [72]. The PANoptosis-related risk score (PANS), a risk score derived from genes associated with PANoptosis, has been proposed. Based on this score, scholars have thoroughly investigated the correlation between PANS and GC prognosis, TME, immunotherapy efficacy, and chemotherapeutic drug sensitivity, offering a more theoretical foundation for anti-tumor therapy [73].

In hepatocellular carcinoma (HCC), a subsequent study explored the expression of PRGs and their prognosis, revealing significant differences in 86.15% (56/65) of PRGs. Among them, 33 PRGs were upregulated in HCC samples (including 20 apoptosis-related genes, 11 pyroptosis-related genes, and 2 necroptosis-related genes), while the remaining 23 showed downregulation (including 7 apoptosis-related genes, 12 pyroptosis-related genes, and 4 necroptosis-related genes). More critically, the expression levels of most PRGs correlated with the overall survival (OS) in patients with HCC [74]. Song et al. predicted treatment response and survival of HCC based on molecular subtyping compatible with PANoptosis and the HPAN-index [75], while Shi et al. and Galluzzi et al. with their respective teams also evaluated a PANoptosis-based prognostic model as a potential prognostic biomarker for HCC patients, and validated the molecular subtypes characterization of the presence of PRGs in HCC [76, 77]. Coincidentally, studies have shown that PANoptosis is closely associated with liver hepatocellular carcinoma (LIHC)-associated survival and immunity, and that the molecular aggregation and prognostic features found in PANoptosis could predict immunological and prognostic conditions of LIHC patients [78].

The role that activation of PANoptosis can play in colon and colorectal cancer has also been further supported in recent studies. In the landscape of genetic variation of PRGs in colon cancer patients, somatic copy number alterations could be found in 19 PRGs, with ZBP1, GSDMD, AIM2, and NLRP3 having the highest copy number variation (CNV). It was further noted that genes including capase-8, MLKL, FADD, and TRADD were upregulated in the tumor samples, while caspase-7, NLRP3, RIPK1, and RIRP3 were downregulated in expression [79]. Another interesting finding comes from NLRP3, one of the key factors mediating the PANoptosis signaling pathway, which has now been shown to be strongly associated with cancer. In experiments on mouse models with colon cancer, researchers found that NLRP3-deficient mice were highly sensitive to dextran sulfate sodium (DSS) and azoxymethane (AOM)-induced colitis-associated cancer (CAC). Mice lacking the inflammasome adaptor protein PYCARD (ASC) and caspase-1 also displayed higher tumor prevalence compared to wild-type mice, and the increased tumor burden was associated with reduced levels of IL-1β and IL-18 at the tumor site [80, 81]. Interferon regulatory factor 1 (IRF1) has also been identified as an upstream regulator of PANoptosis that prevents tumorigenesis in a spontaneous mouse model of CRC by inducing cell death during colitis-associated tumorigenesis [82]. Furthermore, combination therapy with IFN and a nuclear export inhibitor (e.g., KPT-330) has been shown to inhibit tumorigenesis and progression in CRC by inducing ZBP1-dependent PANoptosis through modulation of the regulatory relationship between ADAR1 and ZBP1 [83].

### PANoptosis in genitourinary cancer

A recent study has indicated a strong association between PANoptosis and clear cell renal cell carcinoma (ccRCC). Researchers established a signature model utilizing three specific miRNAs (hsa-miR-21-5p, hsa-miR-223-3p, and hsa-miR-200a-5p) to predict the OS of ccRCC patients. The study revealed that a signature composed of three PANoptosis-related miRNAs could serve as an independent prognostic molecular marker for ccRCC [84].

In the context of prostate adenocarcinoma (PRAD), disturbances and interconnections of PANoptosis, including gene mutations, transcriptional alterations, methylation changes, and clinical characteristics, have also been identified. Through a comparison of multiple PANoptosis pathways enriched differentially between tumors and adjacent normal tissues, Yi et al. demonstrated the interconnectedness of apoptosis, pyroptosis, and necroptosis via a set of genes such as ZBP1, RIPK1, caspase-1, caspase-6, caspase-8, and FADD in PRAD. Consequently, by constructing a PANoptosis signature, accurate predictions can be made regarding the prognosis and immunotherapeutic response of PRAD patients [85].

### PANoptosis in gliomas

The observation of PANoptosis in ischemic brain injury further supports the idea that PANoptosis can serve as a target for the regulation of variety of disorders affecting the central nervous system [86]. Several researchers examined the connection between PANoptosis and gliomas and discovered that PANoptosis has emerged as a significant factor in the prognosis of gliomas. Comparative analysis has revealed genetic and molecular factors associated with prognostic clusters of PANoptosis in gliomas, paving the way for improved prognosis in risk-stratified patient populations based on PANoptosis phenotype [87]. Presently, a machine learning based artificial neural network (ANN) model has been developed for discriminating PANoptosis-related subgroups with drawing implications in predicting prognosis in gliomas [88]. In a study by Song et al., the PANoptotic score was identified as an independent prognostic factor for glioma. The researchers constructed the PANoptotic score using different anti-tumor immunity clinical features based on molecular subtypes of PANoptosome-related genes in gliomas. They observed significant alterations in the expression of five genes included in the PANoptotic score in glioma samples, suggesting a potential intrinsic link between PANoptosis and glioma [89].

### PANoptosis in breast cancer

Increasing evidence suggests that PANoptosis, as a new form of PCD, may be a crucial factor in the development of breast cancer [90], which has led to greater advances in strategies to induce PANoptosis as part of breast cancer treatment. The interactions between PANoptosis and breast cancer can be grouped into three categories: apoptosis induction to promote cell death in breast cancer, synergistic effects of pyroptosis and necroptosis in promoting cell death, and inhibition of apoptosis-induced cell death by necroptosis in breast cancer [91]. He et al. also focused on the relationship between PANoptosis and breast cancer. Through a comprehensive analysis of gene profiles in breast cancer, they determined the percentage of PRGs in each breast cancer cell, suggesting that high expression of PANoptosis is beneficial for reducing the incidence of breast cancer [92]. A recent study constructed and validated a PANoptosis-based prognostic model, which is valuable in predicting the survival outcomes of breast cancer patients. The study further confirmed the importance of PANoptosis-related gene signature in the modulation of TME and drug sensitivity in breast cancer, providing pivotal insights for subsequent mechanical research and personalized treatment decisions [93].

## PANoptosis and TME

### The dynamic interplay between tumor and microenvironment

The relationship between a tumor and its microenvironment is dynamic, as evidenced by the tumor’s influence on the microenvironment and the balance between pro-tumor and anti-tumor factors within it, which collectively regulate tumor growth [94, 95]. The tumor itself can elicit an innate immune response to growth factors, pro-angiogenic factors, and other substances that promote tumor growth and invasion. Chronic inflammation in the TME and the evasion of anti-tumor immune responses are the main factors affecting the occurrence and development of the tumor [96].

TME is a complex network of cellular and non-cellular components within and around a tumor mass, encompassing diverse cell types (such as cancer cells, immune cells, fibroblasts, and endothelial cells), vascular and lymphatic vessels, as well as myeloid and lymphoid elements within tumors. It functions as a dynamic and highly intricate ecosystem that significantly impacts tumor growth, progression, and metastasis, ultimately determining the fate of the tumor and maintaining its equilibrium. Classical theory suggests that the oncogenic mutations of malignant cells lead to the initiation of cancer. Subsequently, the surrounding non-transformed cells are recruited and adapted, accompanied by the release of cytokines, chemokines, and vesicles. The consequences are TME formation and close interaction with cancer cells. It can be assumed that TME is shaped and trained by cancer cells to aid in the development of cancer hallmarks [97]. On one hand, cancer cells can communicate directly with stromal or inflammatory cells in the TME to acquire protective and supportive effects. On the other hand, cancer cells can recruit stromal or immune cells by actively secreting inflammatory and growth factors to induce angiogenesis and lymphogenesis, creating an environment conducive to tumor cell growth, invasion, and evasion of immune surveillance.

Immune cells are actors that play a crucial role in the TME. They coexist with cancer cells and establish intricate interactions with them. The immune microenvironment within tumors is highly complex, with almost all types of immune cells, including macrophages, mast cells, natural killer cells (NKs), dendritic cells (DCs), and T and B lymphocytes, infiltrating cancer tissues [98, 99]. Among these, specific subgroups such as cytotoxic T lymphocytes (CTLs) and NKs can eliminate cancer cells in the TME and impede tumor growth. In solid tumors, tumor-associated macrophages (TAMs) are the most abundant immune infiltrating cells, arising from the local proliferation of resident macrophages and the infiltration of monocyte-derived macrophages. Increased TAM numbers are generally associated with poor prognosis and treatment resistance [100].

### PANoptosis in relation to TME

#### Role of PANoptosis-producing cytokines in the TME

Multiple intracellular components released during PANoptosis process may be involved in the restructuring of the composition and function of the TME, with inflammatory substances having the most notable impact on the TME’s influence and regulation. As inflammatory PCD modes, both pyroptosis and necroptosis release a great deal of inflammatory factors and specific DAMP, inducing the generation of inflammatory reactions. The release of inflammatory factors can further influence the immunogenicity of tumor cells and the activity of immune cells in the TME, ultimately affecting the growth and metastasis of the tumor and playing a pro-tumor or anti-tumor role. Several of the most representative inflammatory mediators, IL-18, IL-1β, IL-1α, and TNF-α, are essential for signaling pathways that cause inflammatory responses. It is reasonable to believe that the release of this end product may contribute to shape the immunological landscape of an inflammatory TME.

IL-18, IL-1β, and IL-1α have a varied role while being part of the IL-1 family. A consensus exists that IL-1 are central mediator of the interactions between cells in the inflammatory TME, both activating the inflammatory responses and orchestrating the diversity of immune responses. The pro-tumorigenic role of IL-1β has been supported for many years, and its association with angiogenesis has also been shown to have a clear correlation in a wide range of tumors, manifested as enhanced angiogenesis and promotion of tumor progression. The role played by IL-18 in TME may be dual in nature. High levels of IL-18 exert pro-tumorigenic effects by participating in angiogenesis, tumor cell invasion, and metastasis. In addition, IL-18 evades the anti-tumor immune response, as reflected by the potential immunosuppressive role of IL-18 in NK cell-controlled tumors. However, IL-18 also displays anti-tumor ability to inhibit the growth and metastasis of many types of tumor cells by activating the immune response of CD4 + T cells and/or NK cells in the TME [101, 102]. Other studies have also proposed that IL-18 is a powerful inducer of type-1 responses in innate and adaptive lymphocytes [103].

IL-1α is likewise a dual-function cytokine. It has been shown that in the TME, dying tumor cells release the inflammatory precursor IL-1α, which, similar to IL-1β, increases tumor invasiveness and angiogenesis [104]. TNF-α is a member of the TNF/TNFR cytokine superfamily and is also a key mediator implicated in inflammation-related cancers. There is substantial evidence that TNF-α can be involved in the promotion and progression of experimental and human cancers. High doses of lo-regional TNF-α can cause hemorrhagic necrosis by selectively destroying tumor vasculature and generating specific T-cell anti-tumor immunity. However, when it is produced in the TME, it acts as an endogenous tumor promoter [105]. In another contradictory finding, based on the fact that the expression of key inflammatory cytokines varies across cancer lineages, Malireddi et al. proposed that activation of TNF-α and IFN-γ could kill cancer cells by inducing PANoptosis [106]. Together, these studies suggest that the effects of the potent inflammatory mediators present in the TME acting on tumors are complex and varied, and that the mechanisms of killing, especially when mediated by PANoptosis, deserve further exploration.

#### Effects of PANoptosis on the tumor immune microenvironment

It is important to note that PANoptosis has been found to impact the immunosuppressive TME [107], which can affect how the PANoptosis signaling pathway regulates cancer in various ways. The roles of apoptosis, pyroptosis, and necroptosis in regulating the immunosuppressive TME and determining clinical outcomes of tumor therapy have been highlighted in recent studies, as well as revealing some mutual crosstalk between the different cell death mechanisms involved in PANoptosis [108]. For example, identified 9 lncRNAs related to PANoptosis and colon adenocarcinoma (COAD) metastasis, which were significantly associated with immune infiltration, indicating that PANoptosis plays a crucial role in the tumor immune microenvironment [109]. More intuitively, it was proposed that the PANoptosis score was significantly correlated with the TME, the infiltration levels of most immune cells (DCs, NKs, CD8+ T cells, and CD4 + T cells), and immune-related genes [67].

Macrophages are a subpopulation of immune cells in the immune system that have shown great moldability in their involvement in immune responses and cancer progression, supported by TME. Macrophages are divided into two main subtypes, M1-like and M2-like. M1 are pro-inflammatory macrophages that act as tumor suppressors by releasing large amounts of pro-inflammatory cytokines (e.g., IL-1β, TNF-α, and IFN-β) accompanied by driving the immune response of powerful T cells and cytotoxic cells. M2-like, on the other hand, exhibit anti-inflammatory properties in order to promote tumor growth and immunosuppression TMEs, including cancer invasion, tumor metastasis, and vascular proliferation [110]. In a study on cervical cancer, scholars found that macrophages were involved in the regulation of immune responses under the common features of immune microenvironment and PANoptosis [111]. A more in-depth study evaluating 33 cancer types in immune cell infiltration found that PANscore was significantly negatively correlated with M1-like macrophages and significantly positively correlated with M2-like macrophages [72]. This suggests that macrophages are not only central players in responding to TME, but also have a reciprocal regulatory relationship with the PANoptosis-mediated tumor immune microenvironment.

DCs are cells that phagocytose dead cells and have a complex interaction with TME in a PANoptotic mechanism. It has long been known that dying cells initiate adaptive immunity by providing antigens to DCs. When tumor cells are undergoing PANoptosis, DCs can recognize specific signaling molecules and phagocytose these signal-carrying cells, presenting tumor antigens to activate other uninfected immune cells, such as T and B cells, directing the onset of immune responses. Effective anti-tumor responses require a subset of tumor-infiltrating DCs, and T-cell-DC crosstalk involving the cytokines IFN-γ, which potentiate CD8+ T-cell-mediated tumor destruction [112]. Early studies have shown that activation of NLRP3 induces adaptive immunity to tumors, and that IL-1β released during this process is required for the priming of IFN-γ-producing, tumor-antigen-specific CD8+ T cells [113]. In a similar vein, a recent study has also elucidated the cytotoxicity of DCs against tumor cell killing. DC-induced-killing is, at least in part, a RIPK1-dependent process, and the elevated phosphorylation of RIPK1 and the increased RIPK1-caspase-8 interaction in target cells also suggest that RIPK1-mediated signals contribute to DC-induced cell death [114]. Thus, the role played by the activation and functional state of DCs on the immune response and tumor development in TME and PANoptosis has been well documented. However, the network of immunosuppressive factors contained in the TME inhibits DC infiltration and suppresses its anti-tumor activity, which in turn modulates the function of DCs in cancer immunity [115]. Further study of the relationship between DCs, TME and PANoptosis could help to gain a deeper understanding of the tumor immune escape mechanism and provide new ideas for the development of tumor immunotherapy strategies.

T cells (primarily CD4+ T, CD8+ T cells, and CTLs) can identify cancer cells throughout the development of cancer and either directly kill cancer cells by releasing cytotoxins, or they can attack cancer cells by stimulating other immune cells. Unique signaling molecules, such as IFN-γ, are released when T cells are triggered by other antigens. This leads to the promotion of PANoptosis, which takes part in the immune response and tumor clearance during the PANoptosis process. CTLs are one of the most specific classes, usually differentiated from CD8+ T cells after activation, and may have more potent cytotoxicity leading to apoptosis or necrosis of tumor cells. In the context of TME, it has been found that the inflammatory mediators released by dying cells are not enough to cross-sensitize CD8+ T cells, and its activation requires RIPK1-mediated NF-κB and intracellular transcription induced by its downstream target genes. This implies that RIPK1-induced NF-κB is a decisive factor in CD8+ T-cell immunity to cell-associated antigens [116]. In addition, researchers have calculated and compared associations between CD8+ T-cell infiltration and the expression of basic markers of distinct types of cell death and several common cancers. They found that the pro-pyroptosis and pro-necroptosis features were broadly linked to greater CD8+ T-cell infiltration [117]. In exploring the role of PANoptosis in pathogenesis and the tumor immune microenvironment of pancreatic cancer (PC), patients were also found to have a significant increase in the infiltration of CD8+ T cells and naive B cells [79]. This further supports the existence of a certain degree of correlation between immune cells, PANoptosis, and TME.

Another special type of cell is the NK cells. NK cells are a group of heterogeneous population, that exhibit cytotoxic, immunomodulatory, tumor cell killing, and IFN-γ-producing properties, and may foster a favorable microenvironment for antigen-specific T-cell immune responses [118]. Through surface receptors, NK cells are able to identify and selectively kill tumor cells. They can also activate other immune cells, strengthening the effect of the combined immune response and increasing the effectiveness of tumor destruction. Chow et al. proposed that NLRP3 inhibits NK cell-mediated tumor metastasis induced by carcinogens. Experimental data indicated that NLRP3 is an important inhibitor of NK cell-mediated control of carcinogenesis and metastasis in the lung TME, manifested by increased activation of NK cells in tumor invasion and enhanced anti-metastasis response. The key point is that NK cells play a greater role in inhibiting tumor metastasis than primary tumors [119]. Furthermore, NK cells have a remarkable role in the battle against HCC. The rate of recurrence of tumor-infiltrating NK cells and circulating NK cells was shown to positively correlate with the survival benefits of HCC, which had prognostic significance [120]. By further constructing a PANoptosis-related gene model of HCC, the new study quantified tumor-infiltrating immune cells using the CIBERSORT algorithm and found that NK cells abundance in the high PANscore group was significantly higher in the TME [76]. RIPK3 has also been shown to modulate NKT cells function through a process independent of the necroptosis pathway, while promoting NKT cell-mediated anti-tumor immune responses [121].

A full understanding of the interaction between PANoptosis and TME helps us to better understand how tumor cells survive and proliferate within the tumors. Investigating the distinct types of immune cells and their ability to mediate anti-tumor or pro-tumor, particularly their underlying mechanisms of interaction with PANoptosis, is crucial. Furthermore, a thorough comprehension of the dynamic processes causing heterogeneity and metabolic plasticity in TME might improve patients’ reactivity to immune-targeted treatment [122]. Implementing targeted interventions to regulate these processes in accordance with changes in the microenvironment is obviously of great significance for the field of tumor therapy research.

## New breakthroughs in cancer therapy: PANoptosis

Cancer remains a daunting challenge, with incidence and mortality rates persistently high and even increasing worldwide. As such, the quest for improved cancer treatment strategies has become an urgent priority.

### Enhancing anti-tumor immunity through PANoptosis: a promising strategy for cancer therapy

**Fig. 3:**
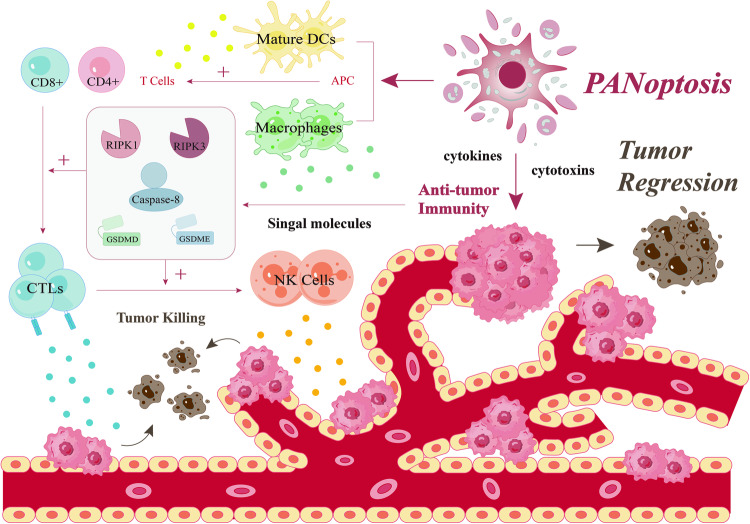
PANoptosis in anti-tumor immunity. With the framework of the TME, PANoptosis promotes tumor regression by inducing potent anti-tumor immunity. Numerous immune cells, including macrophages, DC cells, NK cells, and T lymphocytes, are intimately linked to tumor cell killing. Activation of specific crucial signaling molecules, such as GSDMD, GSDME, Caspase-8, RIPK1, and RIPK3, promotes the activation of immuno stimulators, particularly the CD8+ T cells and NK cells, which release cytokines and cytotoxins to exert potent cytotoxic anti-tumor immunity.

Apoptosis, pyroptosis and necroptosis are some of the most classical types of PCD, which can balance the cell population by regulating cell death, whereas the nature of cancer is the malignant proliferation of cells. Therefore, the promotion of tumor cell death mediated by PANoptosis as a therapeutic means to control cancer growth has become one of the research hotspots for a long time. Within the TME and under the regulation of PANoptosis, anti-tumor immunity determines the final development fate of tumor cells to a certain extent (Fig. 3). On one hand, immune cells present in the TME can play the roles of immune surveillance and immune clearance by inducing tumor cells death; on the other hand, tumor cells can evade PCD by establishing immune tolerance. The majority of studies suggest that PANoptosis plays a beneficial role in cancer by mediating strong anti-tumor immunity. For example, in an experiment discussing the expression characteristics of PANoptosis modulators between normal subjects and gastric cancer (GC) patients, by comparing the immune cell infiltration levels between the two, it was found that patients with GC exhibited greater abundant ratios of naive B cells, naive CD4 T cells, activated memory CD4 T cells, follicular helper T cells, activated NK cells, suggesting that PANoptosis stimulate powerful anti-tumor immunity. Consequently, facilitating PANoptosis could have significant therapeutic implications for GC [123]. Therefore, activating tumor cell-specific death in TME via the PANoptosis pathway can trigger an anti-tumor immune response to enhance the immune function and thus promote tumor regression, providing a promising prospect for cancer therapy.

It has been shown that the programmed death of tumor cells can be directly induced by certain immune cells, or it can participate in a positive feedback loop of anti-tumor immunity by spontaneously triggering a switch from apoptosis to pyroptosis [124]. Since CTLs play a key role in protecting cells from tumors, another, more intuitive example comes from the study of the role of GSDMD, a key regulator in PANoptosis, in CTLs. The results showed that in tumor samples, GSDMD expression was positively correlated with the level of CD8+ T-cell markers in The Cancer Genome Atlas (TCGA) cohorts, and that GSDMD was upregulated in activated CD8+ T cells, while its deficiency would reduce the cytolytic ability of CD8+ T cells. It turns out that GSDMD is a component necessary for anti-tumor immunity by mediating an optimal CTL response to cancer cells [125]. GSDME, as a key member of the apoptosis and pyroptosis crosstalk events, has also been shown to have anti-tumor effects. In a study of 22 cancer-associated GSDME mutations, 20 were found to reduce GSDME function. Indeed, GSDME expression was shown to be suppressed in a variety of cancers (melanoma, colorectal cancer, and triple-negative breast cancer, etc.), and this tumor suppression was also mediated by killer cytotoxic lymphocytes. Not only that, but GSDME expression also enhanced phagocytosis by TAMs as well as infiltration of NK cells, implying that the anti-tumor effects of GSDME are closely related to NK and CD8+ T [126].

Another aspect of PANoptosis that can exert anti-tumor immunity is embodied in RIPK3 and RIPK1, which are both essential components for the activation of necroptosis, and a core part of the interactions between the various PANoptosis signaling pathways. A new study shows that the expression of RIPK3 has a significant positive correlation with populations of tumor immune cells in various tumor types. Therefore, activation of RIPK3, on the one hand can induce TRIM28 in cancer cells to repress and stimulate the increase of immunostimulatory cytokines to enhance the TME. On the other hand, it can contribute to robust cytotoxic anticancer immunity [127]. Concomitantly, ectopic activation of RIPK3 to promote tumor APC loading of tumor antigens is associated with enhanced CD8+ leukocyte-mediated anti-tumor responses [128]. In addition, cross-sensitization of CD8+ T cells in dying cells requires a combination of RIPK1 signaling and NF-κB induced intracellular transcription. When the NF-κB signal is decoupled from necroptosis, the efficiency and tumor immunity are reduced [116]. Cell death mediated by RIPK1 may also increase the activation of infiltrating NK cells and CD8+ T cells and enhance the survival benefit of immune checkpoint blockade [128].

Taken together, these studies underscore the crucial point that inducing PANoptosis in tumor cells can enhance anti-tumor immunity, exerting an effective tumor suppressor effect and providing a feasible approach to improve the efficacy of cancer immunotherapy.

### Targeting PANoptosis for cancer therapy: key signaling targets and their potential therapeutic effects

**Fig. 4:**
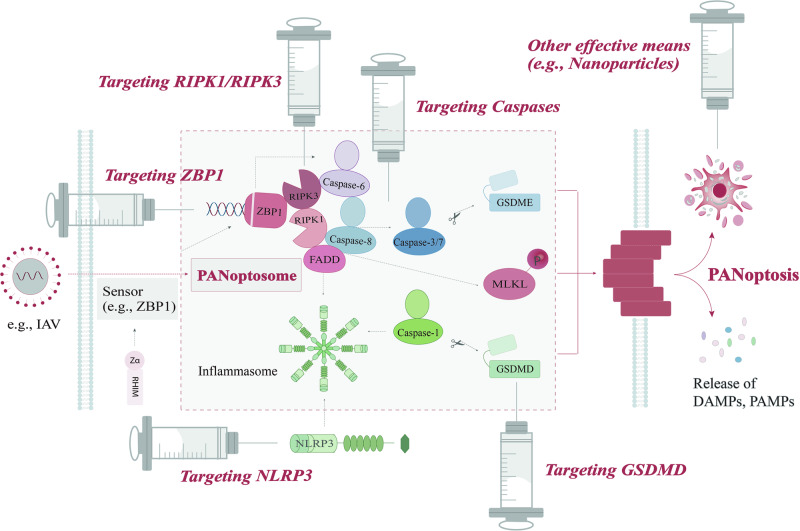
Targeting PANoptosis for cancer therapy. PANoptosis can occur in response to cellular stress or certain microbial infections, such as the IAV. When specific sensors, like ZBP1 are triggered, the assembly of the PANoptosome is formally initiated. This, in turn, promotes the activation of effectors, including the GSDMD/GSDME, caspase-3/7, and MLKL, and ultimately leads to inflammatory cell death as well as the release of DAMPs and PAMPs. The process of PANoptosis is an intricate form of cell death whose mechanism is regulated by several crucial components. These parts come from important members of the apoptosis, pyroptosis, and necroptosis signaling pathways, including ZBP1, the caspase family, NLRP3, RIPK1, and RIPK3. Targeting these regulatory molecules in the PANoptosis signaling pathway is promising for the treatment of certain diseases, especially cancer. Other methods, such as the utilization of nanoparticles to activate PANoptosis, have also been demonstrated to possess therapeutic potential.

We have realized that PCD induced cell death can become one of the important hallmarks of cytotoxic anti-tumor drugs, which can inhibit tumor growth and spread by regulating apoptosis, pyroptosis, or necroptosis signaling pathways [129]. Finding appropriate sensors and agonists or inhibitors of signal molecules in the PANoptosis signaling pathway as targeted drugs is becoming a new approach for cancer therapy. There is already evidence that tumor necrosis factor-α (TNF-α) plus interferon-γ (IFN-γ) was effective against a wide range of tumor cell types, highlighting its potential for clinical application. In diverse cancer lineages (colon and lung cancer, melanoma, and leukemia), the synergistic action of TNF-α and IFN-γ can trigger the activation of a variety of signaling switches such as GSDMD, GSDME, caspase-8, and MLKL. PANoptosis induction of tumor cells in this way has been proven to be an important mechanism for preventing tumorigenesis and inhibiting tumor growth, with targeted therapeutic effects [106]. However, due to its complex mechanism, in addition to its anticancer effect, PANoptosis may also promote the growth of cancer cells under certain conditions. As a simple example, caspase-8 has been reported to have both pro- and anti-tumor effects. On one hand, high expression of caspase-8 in the nucleus of tumor cells prevented typical endogenous apoptosis and induced mitosis, thereby promoting tumor progression. On the other hand, caspase-8 has also been shown to act as a downstream of granzymes, specifically inducing GSDME cleavage and inducing pyroptosis, thus further activating anti-tumor immune response and inhibiting tumor growth [130]. Therefore, it is necessary to find pathways targeting co-regulators in PANoptosis in an attempt to activate one pathway (apoptosis, pyroptosis, or necroptosis) or multiple pathways at the same time for the treatment of cancer (Fig. 4).

#### Targeting ZBP1

As mentioned earlier, ZBP1 serves as an innate immune receptor for IAV infection and is responsible for triggering the assembly of the PANoptosome and inducing PANoptosis upon sensing specific stimuli. Recently, it has been identified as a crucial master switch for PANoptosis and a key signaling node for cell death as it is a co-primary regulator of pyroptosis, apoptosis, and necroptosis [131]. In necrotic tumors, ZBP1 expression is notably elevated, and its deletion impedes tumor necrosis and apoptosis, ultimately inhibiting tumor cell proliferation, suggesting that ZBP1 provides a referenceable drug target for controlling tumor metastasis [132]. In a study exploring the relationship between ADAR1 and ZBP1 in cancer, Zhang et al. described a small molecule, the curaxin CBL0137, which triggered Z-DNA formation in fibroblasts of TME to efficiently activate ZBP1-dependent nuclear necroptosis and strongly reversed immune checkpoint blockade (ICB) unresponsiveness in mouse models of melanoma. Their argument that ZBP1-mediated PCD served as a novel determinant of tumor immunogenicity masked by ADAR1flanks the possibility that ZBP1 has the potential to drive cancer immunity [133]. Therefore, it comes as no surprise that ZBP1 is now considered an important signaling target in tumor therapeutic strategies.

#### Targeting caspases

Caspases are common key proteins in apoptosis and pyroptosis signaling pathways. The centrality of caspases in the regulation of PANoptotic cell death mechanisms makes them unquestionably key targets for cancer therapy. Currently, scholars have identified the potential role of caspase-3-dependent cell death on cancer. In the caspase-3/GSDME signaling pathway, cytotoxic drugs-induced tumor cell death via caspase-3-dependent pyroptosis or apoptosis under the expression of high or low levels of GSDME. By taking this as an entry point, a breakthrough has been made in cancer treatment [134]. Studies have shown that caspase inhibitors modulate radio chemoimmunotherapy-induced cell death in B16 melanoma cells, inducing an anti-tumor immunity in a HMGB1-, nucleotide-, and CD8+ T-cell-dependent manner. In vivo experiments showed that pan-caspase inhibitors such as Zvad-fmk and IDN-6556 (F-03491390) induced apoptosis (and also necroptosis) in a multimodal tumor therapy with radiotherapy (RT), dacarbazine (DTIC), and hyperthermia (HT) combined with drugs, resulting in significant anti-tumor immunity, which was manifested by markedly increased infiltration of DCs and CD8+ T-cell, increased expression of IFN-γ of CD8+ T cells and reduced tumor infiltration of regulatory T cells, which together serve to inhibit tumor growth [135].

#### Targeting NLRP3

Some emerging evidence suggested that NLRP3 may also be a potent target in PANoptotic cancer therapy. The possibility of targeting NLRP3 with novel small molecules to achieve therapeutic effects has been proposed. It was found that activation of NLRP3 in tumors helped to initiate immune responses against tumor cells, which is thought to be a possible effective mechanism for sustained anti-tumor immunity [136]. Another study found that a specific agonist, polyphyllin VI (PPVI), activates the ROS/NF-κB/NLRP3/GSDMD signaling pathway to inhibit non-small cell lung cancer (NSCLC). It exhibits significant anti-tumor properties by preventing cancer cell proliferation. Additionally, given the low sensitivity of chemotherapy in NSCLC, this mechanism also suggests the promise of PPVI as a novel therapeutic drug target [137]. Another example is that chemotherapeutic agent cisplatin (DDP) can work as an anti-tumor agent for triple-negative breast cancer (TNBC), which induce pyroptosis to exert anti-tumor effects in vivo and vitro by up-regulation of the maternally expressed gene 3 (MEG3) and activation of the NLRP3/caspase-1/GSDMD signaling pathway to, thus improving the rate of pathological complete remission in TNBC patients, and providing new sights into the development of strategies for therapeutic interventions in TNBC [138].

#### Targeting RIPK1

Definitive evidence has shown that inducing RIPK1/ RIPK3-mediated necroptosis has the ability to eliminate cancer cells that have acquired resistance to apoptosis. In fact, RIPK1 itself regulates the development of tumors and can be carcinogenic when overexpressed. Wang et al. initially found that RIPK1 was upregulated in TAMs of pancreatic ductal adenocarcinoma (PDA). To further investigate its role in carcinogenic progression, they developed a selective small-molecule inhibitor of RIPK1 and found that RIPK1 promotes differentiation of tolerogenic macrophages in the pancreatic cancer TME, leading to adaptive immune activation and tumor protection. Meanwhile, the inhibition of RIPK1 also enabled PD-1-based immunotherapies effectively enhance the effect of co-stimulatory ligand (ICOS) agonism. Thus, RIPK1 has been described as a checkpoint kinase that controls tumor immunity, and its inhibition can be regarded as a potential tumor therapy [139]. It has also been shown that treatment of mice with the RIPK1-inhibitor necrostatin-1 (Nec-1s) or endothelial-cell-specific deletion of RIPK3 reduced tumor cell-induced endothelial necroptosis and tumor metastasis in mouse models [140]. A few drugs targeting RIPK1 are currently being developed, mainly RIPK1 kinase inhibitors. However, so far, RIPK1 inhibitors have not gained definitive efficacy in clinical trials of human solid tumors [141].

#### Targeting RIPK3

Activation of necroptosis has been shown to respond positively to many chemotherapeutic agents and contribute to chemotherapy-induced cell death. Targeting RIPK3 may offer the advantage of saving cells from a broader spectrum of necroptotic stimuli compared to RIPK1 inhibition [142]. Studies have discovered that RIPK3 expression was often silenced in cancer cells, and its defects were actively selected during tumor growth and development. For example, in 85% of breast cancer patients, the expression of RIPK3 in the tumor was reduced compared with that in normal tissues, and the restoration of RIPK3 expression with hypomethylation agents could promote sensitivity to chemotherapy drugs in a RIPK3-dependent manner and enhance the tumor-killing effect of some chemotherapy drugs. Therefore, it is a better choice for patients with RIPK3-deficient cancers to receive hypomethylating agents to induce RIPK3 expression before receiving conventional chemotherapeutics [143]. As a special point, the expression status of RIPK3 in cervical carcinoma cells has also been shown to influence the efficacy of PolyI:C-induced immunotherapy [144], which facilitates the development of a more rational immunotherapy strategy based on the RIPK3 level.

### Other treatment routes

Immunogenic PANoptosis-initiated cancer sono-immune-reediting nanotherapy has become an eye-catching emerging technology by boosting cancer immune circulation. The study pointed out that the innate or acquired resistance mechanism of refractory tumors to immunotherapies can be effectively served by an immune-reeding anti-tumor strategy based on the engineering EVs-facilitated PANoptotic cell death pathways [145]. Lately, it has been demonstrated that some unique nanoparticles can boost anti-tumor immunity via pyroptosis induction. Zhao et al. designed a biomimetic nanoparticle (BNP) loaded with indocyanine green (ICG) and decitabine (DCT) to photo-activate cancer cell pyroptosis and further secrete pro-inflammatory intracellular contents, hence activating strong systemic anti-tumor immunity to suppress solid and metastatic tumors for primary and distant tumor immunotherapy [146]. Other study has also shown that biodegradable K_3_ZrF_7_:Yb/Er upconversion nanoparticles (ZrNPs), used as pyroptosis inducers for cancer immunotherapy, observably inhibit tumor growth and lung metastasis. In vivo, experiments confirmed that ZrNPs-induced pyroptosis showed excellent anti-tumor immune activity by enhancing DCs maturity and effector-memory T-cell frequency [147].

In addition, Lin et al. found in vivo and in vitro experiments that the absence of cysteine desulfurase (NFS1) synergistically interacted with oxaliplatin treatment could increase the intracellular reactive oxygen species level to trigger PANoptosis, thus significantly enhancing the sensitivity of CRC cells to oxaliplatin. In contrast, prevention of PANoptosis by phosphorylation of NFS1 under oxaliplatin treatment weakened the chemotherapy sensitivity of CRC to oxaliplatin, which improved the anti-tumor efficacy of platinum-based chemotherapy in CRC treatment [148]. Sulconazole also has significant inhibitory effects on a variety of tumor cells, especially in inhibiting the proliferation and migration of esophageal cancer cells. Mechanistically, it has been discovered that sulconazole induced PANoptosis by triggering oxidative stress and inhibiting glycolysis to increase radiosensitivity in esophageal cancer [149]. Therefore, proper regulation of the PANoptosis pathway is also helpful to improve the efficacy of tumor chemotherapy.

## Future directions in cancer treatment

As research into the intricate mechanism of PANoptosis has advanced in recent years, PANoptosis-targeted therapy has emerged as a therapeutic approach with expansive developmental potential. Previous studies have indicated that manipulation of all three PCD pathways—apoptosis, pyroptosis, and necroptosis—can influence cancer progression. Various drugs targeting individual PCD pathways have been developed and demonstrated significant roles in impeding tumor growth. Consequently, PANoptosis, which amalgamates elements from these three pathways, represents a promising breakthrough in anticancer therapy. By selectively targeting and modulating specific molecules within the PANoptosis signaling pathway of tumor cells, PANoptosis-targeted therapy can effectively eliminate cancer cells. Mall et al. identified and validated crucial innate immune biomarkers derived from PANoptosis through pan-cancer transcriptomic profiling, which can serve as therapeutic targets in oncology to enhance the prognosis of cancer patients [150]. Furthermore, compared to traditional chemotherapy, PANoptosis-targeted therapy reduces harm to normal cells, diminishes treatment-related adverse reactions, and enhances treatment specificity. Integrating PANoptosis-targeted therapy with other treatment modalities, such as radiotherapy and immunotherapy, to establish a comprehensive treatment model not only demonstrates broader capacity for inducing cancer cell death but also overcomes drug resistance and better tailors treatment to individual patients. These distinct advantages of PANoptosis-targeted therapy present opportunities to impede tumor growth, augment the effectiveness of current therapies, and improve patient outcomes. With the continuous progress of technology and the accumulation of clinical experience, the development of molecularly targeted drugs in the PANoptotic signaling pathway is poised to become a pivotal focus of new drug development in the future. Precise intervention targeting key proteins in the pathway holds the potential to yield new breakthroughs in cancer treatment.

## Conclusion

PANoptosis represents a form of PCD characterized by the simultaneous activation and interaction of apoptosis, pyroptosis, and necroptosis signaling pathways. It has been established that PANoptosis plays a significant role in the development and advancement of various diseases, particularly cancer. Within the TME, the impact of PANoptosis on tumor progression can vary, influenced by the immune microenvironment and the functions of different pathway components. Therefore, targeting specific molecular elements within the PANoptosis pathways using appropriate strategies holds substantial promise for cancer therapy.

This review focuses on recent research findings concerning key elements of the PANoptosis pathways. Studies indicate that directing interventions towards ZBP1, caspases, NLRP3, RIPK1, and RIPK3 can trigger a robust anti-tumor immune response, facilitating the efficient eradication of cancer cells. Moreover, novel methodologies involving nanoparticles and engineered extracellular vesicles demonstrate potential in harnessing PANoptosis to enhance anti-tumor immunity.

Despite the promising prospects, the dual nature of PANoptosis in influencing tumor growth and progression presents challenges in targeted therapy. Further investigation and clinical trials are essential to validate the safety and efficacy of PANoptosis-targeted treatments. Integrating PANoptosis-targeted therapy with existing modalities is likely to pave the way for personalized and tailored approaches to cancer treatment in the future.
